# Acromegaly in Northeastern Romania: Clinical Characteristics, Therapeutic Management, and Disease Control in a Tertiary Center

**DOI:** 10.3390/life16071093

**Published:** 2026-06-30

**Authors:** Ioana Balinisteanu, Andreea Florea, Maria-Christina Ungureanu, Letitia Leustean, Alexandru Florin Florescu, Stefana Bilha, Lavinia Caba, Roxana Popescu, Lucian-Mihai Antoci, Laura Florea, Eusebiu Vlad Gorduza, Cristina Preda

**Affiliations:** Grigore T. Popa University of Medicine and Pharmacy Iasi, 700115 Iasi, Romania; ioana_florea@d.umfiasi.ro (I.B.); andreea.florea@umfiasi.ro (A.F.); maria.ungureanu@umfiasi.ro (M.-C.U.); letitia.leustean@umfiasi.ro (L.L.); alexandru-florin.florescu@umfiasi.ro (A.F.F.); stefana.bilha@umfiasi.ro (S.B.); roxana.popescu@umfiasi.ro (R.P.); lucian-mihai.antoci@umfiasi.ro (L.-M.A.); laura.florea@umfiasi.ro (L.F.); eusebiu.gorduza@umfiasi.ro (E.V.G.); cristina.preda@umfiasi.ro (C.P.)

**Keywords:** acromegaly, pituitary neuroendocrine tumor, biochemical control, somatostatin receptor ligands, pituitary insufficiency, comorbidities

## Abstract

Acromegaly is a rare chronic endocrine disorder characterized by delayed diagnosis, multisystem comorbidity, and heterogeneous therapeutic response. We aimed to describe the clinical characteristics, tumor profile, treatment patterns, biochemical control, pituitary insufficiencies, and comorbidity burden in an endocrinology tertiary center in northeastern Romania. This observational retrospective study included 87 adult patients admitted for general inpatient evaluation between December 2023 and November 2024, with retrospective data collected from diagnosis and follow-up assessed through the last available hospital visit at St. Spiridon Clinical Emergency Hospital. Clinical, hormonal, imaging, and therapeutic data were analyzed using descriptive statistics and inferential statistical tests. Most patients were diagnosed in middle adulthood, with a female predominance. Macroadenomas and extrasellar extension were common, consistent with advanced tumor stage at presentation. Treatment was predominantly multimodal, with surgery as the main therapeutic intervention and somatostatin receptor ligands as the main medical treatment backbone. Biochemical improvement was observed over time, although complete remission was achieved only in a subset of patients. These findings describe the clinical and therapeutic complexity of acromegaly in a single tertiary-center inpatient cohort and support the need for individualized long-term monitoring.

## 1. Introduction

Acromegaly is a rare chronic endocrine disorder caused by prolonged exposure to excess growth hormone (GH) and the consequent increase in circulating insulin-like growth factor 1 (IGF-1). In most cases, approximately 95%, the disease is caused by a GH-secreting pituitary neuroendocrine tumor, whereas pituitary hyperplasia and extra-pituitary sources are uncommon causes. Although uncommon in the general population, acromegaly has major clinical relevance because of its progressive course, multisystem involvement, and long-term impact on morbidity and mortality [1,2,3,4].

The global burden of acromegaly remains low, with an estimated prevalence of 5.9 cases per 100,000 persons and an annual incidence of 0.38 cases per 100,000 persons [5]. However, the disease is frequently diagnosed late, largely because its clinical manifestations develop insidiously over time. Diagnostic delay has been estimated at a mean of approximately 5.5 years and exceeds 10 years in nearly one-quarter of cases, thereby contributing to advanced tumor stage at diagnosis and to the accumulation of systemic complications [6,7].

Beyond hormonal excess itself, acromegaly is associated with a broad range of comorbidities, particularly cardiometabolic, respiratory, thyroid, osteoarticular, and neoplastic disorders. Large registries and meta-analyses have reported high prevalences of arterial hypertension, diabetes mellitus, sleep apnea syndrome, thyroid nodules, osteoarticular disease, and selected malignancies, emphasizing that the burden of acromegaly extends far beyond endocrine abnormalities alone [8,9,10,11,12,13]. At the same time, pituitary insufficiencies may emerge during the course of disease, particularly after local treatment, and represent an additional source of long-term morbidity [14,15,16,17].

Therapeutic management is typically multimodal and includes surgery, medical therapy, and radiotherapy, used alone or in combination according to tumor characteristics, biochemical activity, and treatment response. Biochemical control is generally assessed using serum IGF-1 together with GH measurements, while imaging and clinical evaluation remain essential for long-term follow-up. Despite advances in treatment, complete remission is not achieved in all patients, and a subset continues to show persistent disease activity or suboptimal response to first-generation somatostatin receptor ligands, supporting the need for individualized treatment strategies [7,18,19,20,21].

In this context, real-world cohort studies remain clinically valuable because they provide integrated information on tumor characteristics, therapeutic pathways, biochemical control, treatment resistance, pituitary function, and associated comorbidities in routine practice. The present study aimed to characterize a real-world cohort of patients with acromegaly from northeastern Romania, with particular focus on clinical profile, tumor features, therapeutic management, disease control, pituitary insufficiencies, comorbidities, and the relationship between tumor size and IGF-1 levels.

## 2. Materials and Methods

### 2.1. Study Cohort and Data Collection

This study was conducted at the Endocrinology Department of St. Spiridon Clinical Emergency Hospital, Iasi, Romania. The cohort was assembled from adult patients with acromegaly who were admitted for general inpatient evaluation between December 2023 and November 2024 and provided informed consent. Day-hospitalization and outpatient-only cases were not included.

The study cohort comprised two categories of patients: (1) individuals with a previously established diagnosis of acromegaly who presented for inpatient follow-up/evaluation, and (2) patients presenting for the first time with clinical features suggestive of acromegaly and subsequently confirmed biochemically.

The diagnosis of acromegaly was established according to the Romanian therapeutic protocol for acromegaly [7], based on the presence of suggestive clinical features in association with characteristic biochemical abnormalities, namely elevated insulin-like growth factor 1 (IGF-1) levels adjusted for age and sex, together with a lack of growth hormone (GH) suppression below 0.4 ng/mL during an oral glucose tolerance test (OGTT). In patients with diabetes mellitus, for whom OGTT was not applicable, a mean GH level > 1 ng/mL over 24 h was considered diagnostic.

Inclusion criteria were age ≥ 18 years, preserved decision-making capacity with provision of informed consent, and absence of exclusion criteria. The exclusion criteria consisted of impaired decision-making capacity or refusal to provide informed consent.

Patients were enrolled between December 2023 and November 2024, according to the date of informed consent. After database review and harmonization, a total of 87 patients were included in the final statistical analysis. Demographic and clinical data, including sex, age at diagnosis, disease duration, and associated comorbidities, were collected retrospectively from the time of diagnosis. Follow-up information was assessed prospectively until each patient’s last available hospital visit at St. Spiridon Clinical Emergency Hospital, which occurred between late 2024 and May 2025 depending on the individual follow-up schedule.

Additionally, variables relevant to the study objectives were standardized and included hormonal parameters (IGF-1 and GH, including OGTT measurements where available), tumor characteristics (maximum tumor diameter, extrasellar extension, imaging findings at diagnosis and at the most recent magnetic resonance imaging evaluation), therapeutic interventions (surgery, type of surgery, radiotherapy and its modality, preoperative medical treatment, and medical treatment at the last visit), and associated comorbidities and complications.

For longitudinal analyses, three standardized time points were defined: at diagnosis when available, during follow-up, and at the last available evaluation.

Missing data were handled using complete-case analysis without imputation, and the number of observations included in each analysis was reported separately in Section 3.

These procedures enabled the construction of a structured database suitable for descriptive analyses, subgroup comparisons, and longitudinal assessments.

### 2.2. Statistical Analysis

Statistical analyses were performed using IBM SPSS Statistics, version 26.

Data were summarized using descriptive statistics, with categorical variables presented as frequencies and percentages, and continuous variables expressed as mean ± standard deviation for approximately normally distributed data, or as median (range) for skewed distributions.

The distribution of continuous variables was assessed using the Shapiro–Wilk test, complemented, where appropriate, by graphical evaluation (e.g., boxplots), including the identification of outliers.

Comparisons between two independent groups were performed using the independent-samples *t*-test when assumptions of normality and homogeneity of variances were met; when the assumption of equal variances was violated, Welch’s correction was applied. For non-normally distributed variables, comparisons between independent groups were conducted using the Mann–Whitney U test.

For paired analyses within the same cohort, the distribution of differences was assessed; when non-normality was present, the Wilcoxon signed-rank test was applied.

Associations between continuous variables with non-normal distributions or susceptibility to outliers were evaluated using Spearman’s rank correlation coefficient (rho), including the analysis of the relationship between maximum tumor size and IGF-1 levels at the first available evaluation.

In cases where extreme values were identified through graphical assessment, sensitivity analyses were performed by temporarily excluding these observations to evaluate the robustness of the observed associations.

For categorical variables assessed at three time points, the Cochran’s Q test was used, followed by McNemar tests for pairwise comparisons.

Statistical significance was defined as *p* < 0.05. Results are presented using the relevant test statistics (t, U, Z, rho, Q) and their corresponding *p*-values, as appropriate.

## 3. Results

### 3.1. Descriptive Demographic Characteristics

**Figure 1:**
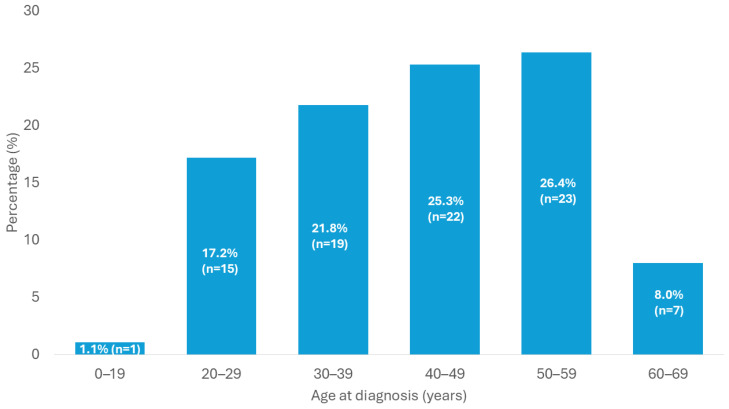
Distribution of patients by age group at diagnosis.

The distribution of the 87 patients according to age group at diagnosis is illustrated in Figure 1. The number of patients in each category was: 1 patient (1.1%) aged 19 years or younger, 15 patients (17.2%) aged 20–29 years, 19 patients (21.8%) aged 30–39 years, 22 patients (25.3%) aged 40–49 years, 23 patients (26.4%) aged 50–59 years, and 7 patients (8.0%) aged 60–69 years. Thus, most patients were diagnosed during middle adulthood, with the largest proportions in the 40–49-year and 50–59-year groups.

The analysis included all 87 patients, with no missing data. Age at diagnosis ranged from 19 to 69 years, with a mean age of 43.11 ± 12.21 years and a median age of 44 years, indicating substantial variability in age at diagnosis, although most patients were diagnosed during mid-adulthood.

Of the 87 patients included in the study, 60 were women (69.0%) and 27 were men (31.0%), indicating a marked female predominance in this cohort.

**Figure 2:**
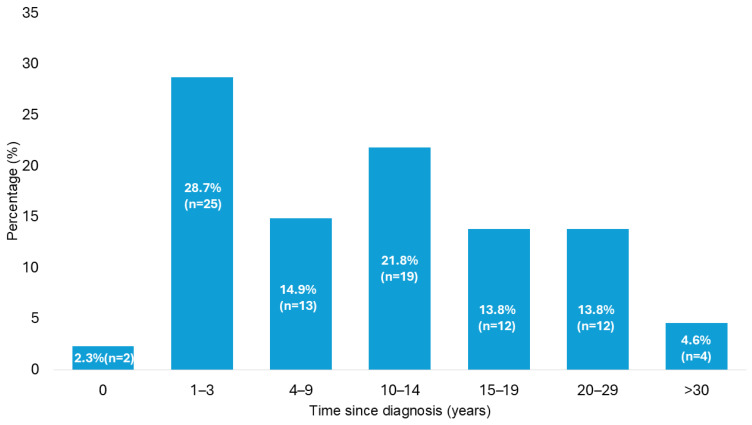
Distribution of patients according to years since diagnosis.

The distribution of disease duration, expressed as years since diagnosis, is shown in Figure 2. The category counts were: 2 patients (2.3%) in the first year after diagnosis, 25 patients (28.7%) with 1–3 years since diagnosis, 13 patients (14.9%) with 4–9 years, 19 patients (21.8%) with 10–14 years, 12 patients (13.8%) with 15–19 years, 12 patients (13.8%) with 20–29 years, and 4 patients (4.6%) with more than 30 years since diagnosis.

These findings highlight the heterogeneous duration of disease evolution within the cohort and reflect the long-term clinical follow-up required in patients with acromegaly.

### 3.2. Therapeutic Management

In the analyzed cohort (87 patients), most patients underwent pituitary surgery. Specifically, 71 patients (81.6%) were operated on, whereas 16 patients (18.4%) did not undergo surgery. This distribution indicates that surgery represented the main therapeutic strategy in the studied cohort.

Among operated patients, the transsphenoidal approach was the predominant surgical technique, being used in 65 patients (91.5% of those who underwent surgery). Transcranial procedures were rare: frontal craniotomy was performed in 1 patient (1.4%), and a fronto-pterional approach in 3 patients (4.2%). In 2 cases (2.8%), a combined approach (transsphenoidal plus fronto-pterional) was used.

Radiotherapy was administered in 42 patients (48.3%), while 45 patients (51.7%) did not receive irradiation. Among irradiated patients, stereotactic radiosurgery with Gamma Knife was the most frequently used modality, being performed in 27 patients (64.3%). Conventional external beam radiotherapy (cobalt therapy) was used in 12 patients (28.6%), while 2 patients (4.8%) received combined treatment with conventional external radiotherapy followed by Gamma Knife. External beam radiotherapy using intensity-modulated radiation therapy with volumetric modulated arc therapy (IMRT-VMAT) was performed in 1 patient (2.4%).

Preoperative medical treatment was administered in 17 patients (23.3% of those with available data), whereas 56 patients (76.7%) did not receive medical therapy before surgery. These data were unavailable for 14 patients (16.1%). Preoperative medical treatment was used selectively, particularly in situations requiring improvement of comorbidities before surgery or reduction in tumor volume to facilitate the surgical approach.

At the last available evaluation, 45 patients (51.7%) were receiving medical treatment for acromegaly, whereas 42 patients (48.3%) were not. Within the latter group, 4 patients were in a therapeutic washout period, 1 patient had an indication for initiation of somatostatin receptor ligand (SRL) therapy at the last evaluation, and 1 patient had temporarily discontinued treatment before Gamma Knife radiotherapy, with the intention to resume it after the most recent hospitalization. In addition, 2 patients were evaluated at the time of diagnosis and had not yet started specific treatment. These findings reflect the heterogeneity of therapeutic status at the last evaluation and indicate that the absence of active medical treatment did not necessarily correspond to disease control in all cases.

The structure of medical treatment at the last available evaluation is presented in Table 1. First-generation somatostatin receptor ligands (SRLs) were the most frequently used treatment class, either as monotherapy or in combination regimens. Monotherapy with first-generation SRLs was recorded in 18 patients (20.7%), while combination regimens including these agents were used in an additional 12 patients, resulting in a total of 30 patients (34.5% of the cohort) exposed to first-generation SRLs.

Dopamine agonists were used as monotherapy in 10 patients (11.5%) and in combination regimens in an additional 12 patients, resulting in a total of 22 patients (25.3%) treated with this class. Second-generation SRLs were used in 6 patients (6.9%) overall, including 3 in monotherapy and 3 in combination regimens. Growth hormone receptor antagonist therapy was prescribed in 4 patients (4.6%), including 1 patient receiving monotherapy and 3 receiving combination treatment.

Overall, these findings show that first-generation SRLs and dopamine agonists formed the backbone of medical therapy in this cohort, whereas second-generation SRLs and growth hormone receptor antagonist therapy were reserved for a smaller subset of patients requiring treatment intensification or combination therapy.

### 3.3. Disease Control and Therapeutic Response

Remission was documented in 20 patients (23.0%). Of these, 11 patients (12.6%) had maintained remission for more than 5 years, whereas 9 patients (10.3%) had a remission duration of less than 5 years. In contrast, 67 patients (77.0%) had not achieved remission at the last available evaluation.

At the last hospitalization, disease control status was evaluated primarily according to GH hypersecretion. Optimal control was achieved in 35 patients (40.2%), while partial response was recorded in another 35 patients (40.2%). Therapeutic inefficacy was identified in 17 patients (19.5%). In patients with hormonal co-secretion, one particular case showed persistent prolactin hypersecretion despite control of the somatotropic axis. This patient had a GH- and prolactin-secreting pituitary macroadenoma complicated by pituitary apoplexy, necrosis of the somatotroph component, and associated somatotroph insufficiency. In this case, disease control status was classified exclusively according to GH secretion.

Symptoms suggestive of active acromegaly were present at the last available evaluation in only 10 patients (11.5%), whereas 77 patients (88.5%) did not show clinically active manifestations. Among the 17 patients (19.5%) classified as having therapeutically ineffective hormonal control, only a subset presented persistent clinical symptoms.

Lack of response to first-generation somatostatin receptor ligands (SRLs) was identified in 18 patients (34.0%) among those exposed to this treatment, whereas 35 patients (66.0%) showed a therapeutic response.

### 3.4. Tumor Characteristics and Extension

Hormonal co-secretion was documented in a relatively small but clinically relevant proportion of the cohort. Most patients, 73 of 87 (83.9%), had a pure somatotroph adenoma. In 14 cases (16.1%), co-secretion of other pituitary hormones was identified, predominantly prolactin: 12 patients (13.8%) had GH-prolactin co-secretion, 1 patient (1.1%) had GH-ACTH co-secretion, and 1 patient (1.1%) had GH-FSH co-secretion.

Extrasellar extension at diagnosis was documented in 50 patients (63.3% of cases with available imaging data), whereas 29 patients (36.7%) showed no extrasellar extension. Imaging data required for assessment of tumor extension at diagnosis were unavailable in 8 patients (9.2%). The high proportion of patients with extrasellar extension suggests that a substantial number of cases were diagnosed at an advanced stage.

Baseline maximum tumor size data were available or classifiable in 76 patients. Imaging data at this time point were unavailable in 11 cases (12.6%). In 2 of the 76 patients, no measurable sellar lesion was identified and these cases were excluded from quantitative tumor-size analyses. In one of these cases, an empty sella appearance was initially documented, followed later by identification of an extrasellar lesion, whereas in the other case the location and type of lesion could not be established by the end of the study, raising the possibility of radiologically occult pituitary hyperplasia. Thus, measurable baseline tumor diameter was included for 74 patients. The median tumor size at diagnosis was 17.0 mm, with values ranging from 3 to 55 mm. Most patients had a pituitary macroadenoma: 60 patients (81.1%) had a maximum tumor diameter of 10 mm or larger, whereas 14 patients (18.9%) had a microadenoma. Giant macroadenomas, defined as lesions with a maximum diameter of 40 mm or larger, were identified in 5 patients (6.8%). Extrasellar tumor extension was present in 62 patients (71.3%), whereas 25 patients (28.7%) had no extrasellar extension.

At the first available evaluation, tumor size data were available for 77 patients, with the same median value of 17.0 mm and a broadly similar overall distribution. The only notable difference was the identification of one additional giant macroadenoma, with a maximum diameter of 57 mm.

Baseline maximum tumor size was compared between patients who subsequently underwent surgery and those who did not. The analysis included 74 patients with measurable tumor size at diagnosis: 62 in the surgery group and 12 in the no-surgery group. Tumor size was larger in the surgery group (median 18.5 mm, IQR 13.0–25.0) than in the no-surgery group (median 6.0 mm, IQR 4.75–14.25). Because of unequal group sizes and skewed distributions, the comparison was performed using the Mann–Whitney U test, which showed a statistically significant difference (U = 129.0 for the no-surgery group versus the surgery group; Z = −3.56; *p* < 0.001).

Maximum tumor size at the last imaging evaluation was compared between patients with and without surgical intervention. This analysis included 85 patients: 15 without surgery and 70 who had undergone surgery. Two patients could not be included in this analysis. In one case, sufficiently late follow-up imaging was not available, while in the other no pituitary tumor could be detected either intrasellarly or extrasellarly by the end of the study, despite persistent clinical and biochemical suspicion.

Imaging at the last evaluation was performed by magnetic resonance imaging in 82 patients and by computed tomography in 3 patients, in situations justified by contraindication to the standard imaging modality or by emergency context, including pituitary apoplexy.

At the last imaging evaluation, patients without surgery had a mean tumor size of 4.33 ± 4.42 mm (median 5.0 mm), whereas those who had undergone surgery had a higher mean tumor size of 8.95 ± 10.95 mm (median 5.0 mm), with markedly greater variability in the operated group. The comparison of maximum tumor size between the two groups was performed using the Mann–Whitney U test, after non-normal distribution had been confirmed by the Shapiro–Wilk test in both groups. No statistically significant difference was observed between patients with and without surgery (U = 446.5; Z = −0.94; *p* = 0.35), although the mean rank was higher in the operated group (44.12 vs. 37.77). These findings indicate that, at the last imaging evaluation, tumor size did not differ significantly between the two groups.

The association between maximum tumor size and IGF-1 level at the first available evaluation was assessed in 69 patients with complete data for both variables. The remaining 18 patients (20.7%) had incomplete data for either tumor size or IGF-1. Both variables showed significant deviation from normality on Shapiro–Wilk testing. Maximum tumor size at the first available evaluation showed a non-normal distribution (W = 0.908, *p* < 0.001), and IGF-1 levels were also non-normally distributed (W = 0.945, *p* = 0.005). Accordingly, the association was assessed using Spearman’s rank correlation coefficient.

Spearman correlation analysis revealed a weak but statistically significant positive correlation between maximum tumor size and IGF-1 level (rho = 0.240, *p* = 0.047; N = 69). This finding suggests that larger tumors tended to be associated with higher IGF-1 levels at the first available evaluation, although the strength of this relationship was limited.

### 3.5. Hormonal Profile and Sex-Based Comparisons

Hormonal profile comparisons are summarized in Table 2. GH levels measured during the oral glucose tolerance test (OGTT) did not differ significantly between women and men, either at diagnosis or at the first available evaluation. Similarly, although IGF-1 levels tended to be higher in men than in women at both time points, these differences did not reach statistical significance.

By contrast, the longitudinal analysis of IGF-1 showed a statistically significant decrease between the first available evaluation and the last evaluation. Most patients showed lower IGF-1 values at the last evaluation, with only a small number showing higher or unchanged values. The large effect size indicates a substantial reduction in IGF-1 over time in the analyzed cohort.

### 3.6. Comorbidities and Associated Complications

Comorbidities were frequent and heterogeneous in this cohort of 87 patients with acromegaly, reflecting the multisystemic nature of the disease. Their distribution is summarized in Table 3. For sex-specific conditions, percentages were calculated within the corresponding subgroup, namely women for gynecological disorders and men for benign prostatic adenoma/hyperplasia.

The most frequent comorbidities were dyslipidemia, present in 54 patients (62.1%), nodular goiter in 48 patients (55.2%), arterial hypertension in 47 patients (54.0%), obesity in 42 patients (48.3%), and ocular disorders in 40 patients (46.0%). Disturbances of glucose metabolism were also common, being documented in 37 patients (42.5%) at the last available evaluation, including diabetes mellitus in 24 patients (27.6%) and prediabetes in 14 patients (16.1%).

Data on glucose metabolism were also available at diagnosis. At that time, diabetes mellitus was present in 12 patients (13.8%) and impaired fasting glucose/prediabetes in 3 patients (3.4%), whereas 72 patients (82.8%) had no documented abnormalities of glucose metabolism. By the last available evaluation, the number of patients with diabetes mellitus had increased to 24 (27.6%), and the number with prediabetes to 14 (16.1%). Among the 24 patients with diabetes mellitus at the last evaluation, 8 patients (33.3%) required insulin therapy, whereas 16 (66.7%) were managed with other treatment regimens.

Ocular involvement was documented in 40 patients (46.0%), with heterogeneous underlying mechanisms. In 13 patients, ocular abnormalities were potentially related to pituitary tumor compression and included optic nerve atrophy, peripapillary atrophy, and visual field defects, such as hemianopia and arcuate or paracentral scotomas. In the remaining 27 cases, ocular pathology was attributed mainly to vascular-metabolic or degenerative conditions, including hypertensive retinopathy, diabetic retinopathy, retinal angiosclerosis, age-related cataract, primary open-angle glaucoma, age-related macular degeneration, and other retinal or lens abnormalities.

Digestive comorbidities were also frequent and included gallbladder lithiasis in 32 patients (36.8%), other gastroenterological disorders in 28 patients (32.2%), and hepatic steatosis in 21 patients (24.1%). Other gastroenterological disorders covered a broad, predominantly benign clinical spectrum. Anorectal involvement was the most frequent, mainly represented by hemorrhoidal disease. Chronic liver disease was present in 6 patients and included chronic viral hepatitis B or C, as well as compensated cirrhosis of viral and/or metabolic etiology. Benign liver lesions, mainly hepatic hemangiomas, were identified in 3 patients. Gastric and esophageal disorders, including chronic gastritis, Helicobacter pylori-associated gastritis, reflux esophagitis, and gastroduodenitis, were documented in 4 patients. Functional bowel disorders, such as irritable bowel syndrome, were present in 2 patients. Isolated diagnoses also included chronic pancreatitis and chronic acalculous cholecystitis. Colorectal neoplasms are detailed separately under the neoplasia category.

Musculoskeletal or orthopedic involvement was reported in 26 patients (29.9%) and was frequently associated with the osteoarticular changes characteristic of acromegaly. Manifestations were predominantly degenerative and included gonarthrosis, coxarthrosis, cervico-dorso-lumbar spondylosis, spondylodiscarthrosis, and disc disease, often in multisegmental combinations. In some patients, advanced forms were documented, complicated by fractures, femoral head osteonecrosis, or requiring arthroplasty. Associated conditions such as carpal tunnel syndrome, scapulohumeral periarthropathy, and polyarthrosis were also recorded.

Sex-specific comorbidities were analyzed separately. Gynecological disorders were present in 18 of 60 women (30.0%), while benign prostatic adenoma or hyperplasia was documented in 8 of 27 men (29.6%). Gynecological pathology was predominantly benign and included uterine fibromatosis, ovarian cysts, and benign breast lesions, with isolated cases of ovarian cystic teratoma, endometrial polyp, and other benign uterine tumors.

Bone involvement included osteopenia in 17 patients (19.5%) and osteoporosis in 13 patients (14.9%). In addition to arterial hypertension, cardiovascular comorbidities included arrhythmias in 12 patients (13.8%) and ischemic history in 11 patients (12.6%). Among ischemic events, chronic ischemic heart disease was the most frequent, while cerebrovascular ischemic disease and peripheral arterial disease were less common. Arrhythmias were heterogeneous, including atrial fibrillation or flutter, supraventricular arrhythmias, ventricular extrasystoles, bigeminy, and non-sustained ventricular tachycardia. In selected cases, interventional treatment such as radiofrequency ablation or pacemaker implantation was required. Severe aortic involvement was documented in 4 patients (4.6%) and included one case of operated aortic dissection, one case of ascending aortic ectasia, and valvular aortic disease in the remaining cases.

Sleep apnea syndrome was identified in 14 patients (16.1%), predominantly in obstructive forms. Severe obstructive sleep apnea was the most frequent subtype, followed by moderate and mild obstructive forms. Mixed sleep apnea was diagnosed in 2 patients, and severe central sleep apnea associated with restrictive ventilatory dysfunction was identified in 1 patient. In 3 cases, the exact subtype could not be specified.

Regarding thyroid disease, in addition to nodular goiter, autoimmune thyroiditis was present in 11 patients (12.6%), primary hypothyroidism in 8 patients (9.2%), and papillary thyroid carcinoma in 3 patients (3.4%). Overall, neoplasms were documented in 6 patients (6.9%), including 3 cases of papillary thyroid carcinoma, 2 colorectal malignancies, and 1 laterocervical basal cell carcinoma.

Less frequent comorbidities included chronic infectious diseases in 10 patients (11.5%), renal involvement in 8 patients (9.2%), hernias in 8 patients (9.2%), adrenal adenoma in 7 patients (8.0%), bradycardia in 6 patients (6.9%), hepatic cysts in 6 patients (6.9%), dolichocolon in 5 patients (5.7%), disc herniation in 5 patients (5.7%), other intracranial abnormalities in 5 patients (5.7%), renal lithiasis in 5 patients (5.7%), severe aortic involvement in 4 patients (4.6%), colonic diverticulosis in 4 patients (4.6%), reactive hypoglycemia in 3 patients (3.4%), nasal septum deviation in 3 patients (3.4%), parathyroid disease in 3 patients (3.4%), colonic polyps in 2 patients (2.3%), pituitary apoplexy in 2 patients (2.3%), epilepsy in 2 patients (2.3%), and intestinal obstruction in 2 patients (2.3%).

Chronic infectious diseases were predominantly represented by chronic viral hepatitis, but also included tuberculosis, HIV infection, and isolated latent chronic infections. Renal involvement had heterogeneous etiologies, including chronic kidney disease, polycystic kidney disease, focal renal lesions, and renal ptosis. Other intracranial abnormalities, distinct from the pituitary adenoma, included meningiomas, communicating hydrocephalus, an intradiploic occipital nodular lesion, and a ruptured anterior communicating artery aneurysm treated by embolization. Parathyroid disease included parathyroid hyperplasia, primary hyperparathyroidism due to parathyroid adenoma, and a non-secreting parathyroid adenoma.

Additional isolated medical antecedents, each recorded in a single patient and not included in the quantitative analysis, were documented descriptively. These included a mediastinal mass, bilateral sensorineural hearing loss, chronic ENT disorders, Raynaud syndrome, dermatologic conditions, pancreatic cystic lesions, generalized lipomatosis, bilateral hydrocele, and toxic thyroid disease.

### 3.7. Pituitary Function: Insufficiencies and Temporal Dynamics

Pituitary insufficiencies were analyzed dynamically at three main time points—at diagnosis, during follow-up, and at the last available evaluation—in order to capture the impact of both the pituitary tumor and therapeutic interventions on endocrine function. For longitudinal categorical testing with Cochran’s Q and McNemar tests, gonadotropic and corticotropic insufficiencies were treated as binary variables, with both complete and partial forms coded as present.

The prevalence of pituitary insufficiencies across the three time points is summarized in Table 4. At diagnosis, pituitary insufficiencies were relatively uncommon: thyrotropic and gonadotropic insufficiency were each present in 9 patients (10.3%), while complete corticotropic insufficiency was identified in 3 patients (3.4%) and partial corticotropic insufficiency in 1 patient (1.1%). Somatotropic insufficiency was documented in only 1 patient (1.1%), and diabetes insipidus was not present in any patient at diagnosis.

During follow-up, the prevalence of pituitary insufficiencies increased substantially. Thyrotropic insufficiency was documented in 32 patients (36.8%), while complete gonadotropic insufficiency was observed in 28 patients (32.2%), together with 2 additional cases of partial gonadotropic insufficiency (2.3%). Corticotropic impairment also became more frequent, with 16 patients (18.4%) presenting complete insufficiency and 13 patients (14.9%) partial insufficiency. Somatotropic insufficiency was recorded in 4 patients (4.6%), while diabetes insipidus occurred in 10 patients (11.5%), most likely in the postoperative setting.

At the last available evaluation, the profile of pituitary deficits appeared relatively stable, with persistently high prevalences of thyrotropic, gonadotropic, and corticotropic insufficiency. Thyrotropic insufficiency was present in 30 patients (34.5%), complete gonadotropic insufficiency in 32 patients (36.8%), partial gonadotropic insufficiency in 1 patient (1.1%), and corticotropic insufficiency in 28 patients (32.2%), including 15 patients (17.2%) with complete and 13 patients (14.9%) with partial forms. Somatotropic insufficiency remained present in 4 patients (4.6%), unchanged from the intermediate evaluation, whereas diabetes insipidus persisted in only 2 patients (2.3%). At the last evaluation, among the 33 patients with gonadotropic insufficiency, only 11 (33.3%) were receiving hormone replacement therapy.

Statistical testing confirmed these temporal trends. For the thyrotropic axis, Cochran’s Q test showed significant differences across the three time points (Q = 38.96; df = 2; *p* < 0.001). Post hoc McNemar testing with Bonferroni correction demonstrated a significant increase in prevalence between diagnosis and follow-up (*p* < 0.001), as well as between diagnosis and the last evaluation (*p* < 0.001), with no significant difference between follow-up and the last evaluation (*p* = 0.500).

For the gonadotropic axis, considering complete and partial insufficiency together, Cochran’s Q test also showed significant differences across the three evaluations (Q = 38.00; df = 2; *p* < 0.001). Post hoc McNemar analysis showed a significant increase between diagnosis and follow-up (*p* < 0.001) and between diagnosis and the last evaluation (*p* < 0.001), whereas no significant difference was observed between follow-up and the last evaluation (*p* = 0.375).

For the corticotropic axis, again considering complete and partial forms together, Cochran’s Q test demonstrated a significant overall difference across the three time points (Q = 48.08; df = 2; *p* < 0.001). McNemar testing showed a significant increase between diagnosis and follow-up (*p* < 0.001) and between diagnosis and the last evaluation (*p* < 0.001), with no significant difference between follow-up and the last evaluation (*p* = 1.000).

By contrast, somatotropic insufficiency remained rare at all time points. Cochran’s Q test showed a borderline overall difference (Q = 6.00; df = 2; *p* = 0.050), but post hoc McNemar analyses did not reveal significant differences in any pairwise comparison (all *p* > 0.20).

Diabetes insipidus showed a distinct pattern characterized by postoperative occurrence and partial remission. No patient had diabetes insipidus at diagnosis, whereas 10 patients (11.5%) developed it during follow-up, mainly in the postoperative setting, and only 2 patients (2.3%) still had persistent diabetes insipidus at the last evaluation. Cochran’s Q test showed significant differences across the three time points (Q = 16.80; df = 2; *p* < 0.001), while McNemar analysis demonstrated a significant increase between diagnosis and follow-up (*p* = 0.002), followed by a significant decrease between follow-up and the last evaluation (*p* = 0.008), with no significant difference between diagnosis and the last evaluation (*p* = 0.500).

Overall, these findings indicate a clear increase in the prevalence of pituitary insufficiencies after diagnosis, followed by relative stabilization at the last evaluation. The gonadotropic and thyrotropic axes were the most frequently affected, followed by the corticotropic axis, whereas somatotropic insufficiency and diabetes insipidus were less frequent and occurred predominantly as post-therapeutic events.

Remission of pituitary insufficiencies was analyzed only in patients who had developed at least one axis deficit during the disease course. Of the 46 patients with documented pituitary insufficiency, 3 patients (6.5%; 3.4% of the entire cohort) showed remission of one or more pituitary axes over time, whereas in 43 patients (93.5%) the insufficiency remained persistent. All three remission cases occurred in women and were documented in the postoperative setting. In one case, thyrotropic and corticotropic insufficiency that developed after a second transsphenoidal intervention later resolved after several years of hormone replacement and treatment with first-generation somatostatin receptor ligands. In the other two cases, thyrotropic and gonadotropic insufficiency present at diagnosis resolved after transsphenoidal tumor resection. These observations suggest that although pituitary insufficiency is usually persistent, endocrine recovery may occur in a small subset of patients during long-term follow-up.

## 4. Discussion

### 4.1. Main Findings

The analyzed cohort represents a single tertiary-center inpatient population from northeastern Romania. Age at diagnosis was concentrated in middle adulthood (mean 43.11 years; median 44 years). This profile is close to the LAS database, which reported a median age at diagnosis of 45.2 years, and within the middle-adulthood range described by the Danish nationwide cohort, which reported a mean age at diagnosis of 49.3 years [9,11]. The female predominance observed in the present cohort (69.0%) should be interpreted cautiously rather than as a stable epidemiological feature, because the LAS database reported only a slight female predominance (54.5%) and the Danish nationwide cohort reported an almost balanced distribution (51% female) [9,11]. Thus, the sex distribution in the present series may reflect local referral pathways, inpatient selection, and the inclusion of both prevalent and newly diagnosed cases.

The tumor profile, dominated by macroadenomas, extrasellar extension, and a subset of giant macroadenomas, is also compatible with the substantial tumor burden reported in large acromegaly cohorts. In the present cohort, macroadenomas accounted for 81.1% of measurable baseline tumors, the median tumor diameter at diagnosis was 17.0 mm, and giant macroadenomas were identified in 6.8% of patients with measurable baseline tumor size. Macroadenomas accounted for 71.8% of cases in the LAS database and 77% of cases in the Danish nationwide cohort, with median tumor sizes of 15 mm and 16 mm, respectively [9,11]. The present cohort therefore fits the broader pattern of frequent macroadenoma at diagnosis, but the high extrasellar-extension burden documented at diagnosis in 63.3% of patients with available imaging data should be compared cautiously because the available cohort data do not provide an identical extrasellar-extension variable. The closest verified comparator is tumor invasion in the LAS database, reported in 47.6% of baseline tumors [9]. The newly added baseline comparison by surgical status further supports the clinical impression that patients who underwent surgery had larger tumors at diagnosis than those managed without surgery (median 18.5 mm versus 6.0 mm, *p* < 0.001); however, this finding should be interpreted within the center-specific treatment pathway and not as a generalizable estimate of surgical selection in all patients with acromegaly.

Therapeutic management in this cohort was predominantly multimodal, with surgery as the main intervention (71/87 patients, 81.6%), frequent use of radiotherapy (42/87 patients, 48.3%), and long-term medical therapy centered on first-generation SRLs (30/87 patients, 34.5% of the cohort). The surgery-centered structure is consistent with the Danish nationwide cohort, in which 76–83% of incident patients received primary pituitary surgery within five years of diagnosis, although the need for secondary surgery decreased over time from 13% in the 1990s to 7% in the 2000s and 5% in 2010–2021 [11]. Radiotherapy should be interpreted more cautiously: it was used in 9% of incident patients within five years of diagnosis in the Danish cohort, while the multicenter stereotactic radiosurgery cohort mainly included patients with prior surgical resection (93.0%) and residual or recurrent tumor (87.9% and 5.1%, respectively) [11,17]. Therefore, the frequent use of radiotherapy in the present cohort likely reflects tertiary referral, advanced tumor burden, residual or difficult-to-control disease, and selection of patients requiring extensive inpatient evaluation, rather than a generalizable estimate of radiotherapy use. Regarding medical therapy, SSA initiation in the Danish cohort increased from 32% in the 1990s to 59% in the 2000s and 57% in 2010–2021, and current treatment reviews describe SRLs as a central medical option for persistent disease after surgery or for selected patients managed primarily with medical therapy [11,21]. This supports interpreting first-generation SRLs as the main medical backbone in the present cohort, while avoiding direct generalization of local treatment proportions.

The distinction between strict remission and optimal or partial biochemical control remains important. Although remission was documented in only a subset of patients (20/87 patients, 23.0%), longitudinal IGF-1 values decreased significantly, and many patients reached either optimal control (35/87 patients, 40.2%) or partial response (35/87 patients, 40.2%) at the last available hospitalization. Conversely, therapeutic inefficacy persisted in 17/87 patients (19.5%), and lack of response to first-generation SRLs was identified in 18 patients (34.0% of exposed patients). These findings should therefore be interpreted as evidence of meaningful biochemical improvement in a difficult-to-control cohort, rather than as proof of uniformly definitive disease remission.

The clinically relevant proportion of lack of response to first-generation SRLs (18 patients, 34.0% of those exposed) is compatible with known biological heterogeneity in acromegaly. Sparsely granulated adenomas and tumors with lower SSR2 expression tend to respond less favorably to first-generation SRLs, whereas SSR5 expression may support the efficacy of pasireotide [19,20,21]. Although histopathological and receptor-expression stratification was not systematically available in the present cohort, the clinical pattern supports the need for individualized therapeutic escalation and provides a rationale for further molecular and genetic characterization.

The imaging analyses also require a treatment-aware interpretation. Baseline tumor size was significantly larger in patients who subsequently underwent surgery (median 18.5 mm versus 6.0 mm, *p* < 0.001), whereas maximum tumor size at the last imaging evaluation did not differ significantly between operated and non-operated patients (median 5.0 mm versus 5.0 mm, *p* = 0.35). The latter result may reflect the combined effects of surgery, radiotherapy, postoperative tissue changes, residual tumor burden, and unequal group sizes, rather than an absence of baseline clinical differences between the groups.

The weak but statistically significant positive correlation between maximum tumor size and IGF-1 level at the first available evaluation (rho = 0.240, *p* = 0.047; N = 69) is biologically plausible but should not be overinterpreted. Tumor size may contribute to biochemical severity, but IGF-1 levels are also influenced by secretory activity, tumor biology, individual variability, prior therapy, and timing of evaluation. Thus, tumor diameter is useful for clinical characterization but is not sufficient as an individual predictor of hormonal excess.

In the present cohort, the comorbidity profile largely overlaps with that reported in major registries and meta-analyses, although with some particularities reflecting both the timing of evaluation, namely the last visit rather than diagnosis, and the local clinical context. The prevalences of dyslipidemia (62.1%), arterial hypertension (54.0%), obesity (48.3%), and glucose metabolism disturbances (42.5%, with diabetes mellitus in 24 patients, 27.6%, and prediabetes in 14 patients, 16.1%) define a pronounced cardiometabolic burden. By comparison, in the Liege Acromegaly Survey (LAS) database, diabetes mellitus is reported at diagnosis in 27.5% of patients and hypertension in 28.8%, while the Slagboom meta-analysis describes hypertension in 38% and glucose metabolism disturbances in approximately 34% of patients. The fact that, in the present cohort, these prevalences at the last evaluation are higher than those generally reported at diagnosis and very close to those observed during follow-up in longitudinal cohorts such as Acro_DEN, where hypertension was present in 49% and type 2 diabetes in 26%, supports the concept of progressive accumulation of cardiometabolic risk over time, as also suggested by Danish data and by the study of Carmichael, in which patients with incomplete biochemical control had higher frequencies of hypertension and diabetes [9,10,11,12].

The osteoarticular and bone profile of our cohort also fits this pattern of cumulative burden described in longitudinal studies. Musculoskeletal/orthopedic involvement was present in 29.9% of patients, a figure very close to the prevalence of joint involvement reported in the Acro_DEN cohort (31%), where the risk of articular disease increases significantly in more recently diagnosed patients. Osteopenia (19.5%) and osteoporosis (14.9%) in our cohort are slightly higher than the prevalence of osteoporosis reported in LAS (12.3% at diagnosis) and in Acro_DEN (approximately 11% during follow-up), which may reflect the mean age of the cohort, disease duration, and the cumulative impact of hypogonadism, treatments, and other risk factors. Overall, these findings support recent literature indicating that osteoarticular comorbidities are not confined to the time of diagnosis, but may continue to worsen or become more apparent over time, even in patients who achieve hormonal control [9,11].

Nodular thyroid disease was very frequent in our cohort, with nodular goiter present in 55.2% of patients, a prevalence higher than that reported in LAS (34.0%) and above the pooled prevalence of 44% reported in the Slagboom meta-analysis for thyroid nodules at diagnosis. This difference may reflect a higher degree of thyroid screening, such as systematic ultrasound evaluation, as well as regional factors, including iodine status and the high prevalence of thyroid nodules in the Romanian population [9,10].

On the other hand, sleep apnea syndrome was identified in 16.1% of patients, a value slightly lower than the prevalence reported in LAS (25.5% at diagnosis), but very close to that reported in the Danish cohort during follow-up (16%). Given that the diagnosis of sleep apnea requires dedicated investigations, partial underdiagnosis in our cohort is likely. In addition, an important proportion of patients in this cohort were exposed to somatostatin analog therapy, including preoperatively or long term, which, according to literature data, may contribute to improvement of obstructive sleep apnea by reducing the volume of pharyngeal soft tissues and pituitary tumor mass. In this context, the observed prevalence of sleep apnea in our cohort should be interpreted in light of both possible underdiagnosis and the favorable effect of hormonal control, particularly SRL therapy, on respiratory manifestations, while the distribution of subtypes, predominantly obstructive and of moderate-to-severe intensity, remains in agreement with published data [9,11].

Regarding neoplasms, these were identified in 6.9% of patients, with papillary thyroid carcinoma (3.4%) and colorectal neoplasia (2 cases) predominating, together with one case of basal cell carcinoma. Although the relatively small sample size does not allow robust estimation of relative risk, the pattern of tumor localization is consistent with the meta-analysis by Xiao, which showed a moderately increased overall cancer risk in acromegaly (SIR approximately 1.45), with marked increases for thyroid cancer, colorectal cancer, and central nervous system tumors. The predominance of papillary thyroid carcinoma and the presence of colorectal malignancies support alignment of our cohort with the oncologic profile described in the literature, even if the absolute number of cases is limited by sample size. In addition, the frequent association of metabolic and cardiovascular comorbidities with acromegaly, as reflected both in our data and in the LAS, Slagboom, Acro_DEN, and Carmichael studies, underscores the need for an integrated approach aimed not only at hormonal control, but also at prevention and active long-term management of comorbidities [9,10,11,12,13].

In the analyzed cohort, pituitary insufficiencies showed a clear progressive evolution compared with diagnosis: initially they were relatively uncommon, with complete thyrotropic insufficiency in 10.3%, complete gonadotropic insufficiency in 10.3%, cumulative corticotropic insufficiency in 4.6%, including 3.4% complete and 1.1% partial forms, somatotropic insufficiency in 1.1%, and no diabetes insipidus at diagnosis. Subsequently, a marked increase was observed during follow-up and at the last evaluation, with predominance of the thyrotropic, gonadotropic, and corticotropic axes. At the last evaluation, complete thyrotropic insufficiency was present in 34.5%, total gonadotropic insufficiency in 37.9%, including 36.8% complete and 1.1% partial forms, and total corticotropic insufficiency in 32.1%, including 17.2% complete and 14.9% partial forms. This profile suggests that hypopituitarism becomes a major clinical component in the course of acromegaly, mainly as a cumulative result of local therapeutic interventions, particularly surgery and, in a subgroup, radiotherapy, rather than as a dominant expression of the disease at diagnosis.

In the acromegaly literature, the meta-analysis comparing conventional fractionated radiotherapy with stereotactic radiosurgery identifies hypopituitarism as the most frequent late complication, with a pooled mean prevalence of approximately 33% after conventional fractionated radiotherapy versus approximately 22% after stereotactic radiosurgery. The higher values observed at the last evaluation in the present cohort for pituitary axis deficits, namely thyrotropic 34.5%, gonadotropic 37.9%, and corticotropic 32.1%, are plausibly explained by the fact that the present analysis quantifies cumulative deficits throughout the disease course and explicitly includes partial forms in a population with mixed therapeutic exposure, whereas radiotherapy meta-analyses often focus on newly developed hypopituitarism after irradiation [14,15].

Published data on the distribution of pituitary axis deficits after radiosurgery generally suggest predominant involvement of the thyrotropic axis, followed by the gonadotropic, corticotropic, and somatotropic axes. This trend is described both in the meta-analysis by Albano et al., reporting newly developed hypopituitarism with cumulative estimates of approximately 20–28% for secreting adenomas, and in the multicenter International Gamma Knife Research Foundation study in acromegaly, where newly developed endocrinopathies after radiosurgery were dominated by thyrotropic deficiency (16.7%), followed by gonadotropic deficiency (13.2%) and corticotropic insufficiency (10.5%). In the present cohort, at the last evaluation, the frequency ranking was slightly different, with gonadotropic insufficiency being the most frequent (37.9%), followed very closely by thyrotropic insufficiency (34.5%), and then corticotropic insufficiency (32.1%), while somatotropic insufficiency remained rare (4.6%). This difference in hierarchy is probably explained by the fact that the present results reflect the prevalence of deficits at the end of a disease course involving cumulative therapeutic interventions, especially surgery with or without radiotherapy, whereas radiosurgery studies mainly summarize newly developed insufficiencies after irradiation. At the same time, the close numerical proximity between gonadotropic and thyrotropic insufficiency in the present cohort suggests a broadly comparable pattern to that described in the post-radiosurgery literature, in which these two axes are generally the most frequently affected, while the somatotropic axis remains the least commonly deficient [16,17].

Diabetes insipidus showed a distinct behavior, suggestive of a predominantly post-therapeutic and often transient event: it was absent at diagnosis, present in 11.5% during follow-up, and persistent in only 2.3% at the last evaluation. In the multicenter International Gamma Knife Research Foundation study, diabetes insipidus was present before radiosurgery in 1.1%, and no new diabetes insipidus was observed after radiosurgery, supporting the view that this complication is more closely associated with pituitary surgery and the postoperative period than with stereotactic radiosurgery [17].

Overall, the present findings support the view that this cohort represents a profile of acromegaly frequently diagnosed at advanced tumor stages, with an important proportion of patients requiring adjuvant treatment and long-term medical therapy, in a context where complete biochemical control and sustained remission remain difficult to achieve in a subset of patients. At the same time, the significant reduction in insulin-like growth factor 1 and the high proportion of patients with either optimal control or partial response suggest an overall effective management strategy, although one that requires continuous individual adjustment.

### 4.2. Limitations and Methodological Considerations

The interpretation of these findings should take into account the inherent limitations of a clinical database, including retrospectively collected elements, missing data for certain variables, and non-uniform timing of the last available hospital visit. The cohort was derived from a single tertiary endocrinology center and included only consenting adults admitted for general inpatient evaluation; day-hospitalization and outpatient-only cases were not included. This design may have selected patients requiring more extensive evaluation and limits the generalizability of the findings to other Romanian institutions.

Selection and survivorship bias should also be considered. Patients who died before enrollment, were followed elsewhere, had very mild disease, or had stable/remitted disease not requiring inpatient evaluation were not captured. Because the prospective follow-up period was short and all included patients were already in active hospital care, reliable categories of regular follow-up versus lost to follow-up could not be defined, and a survivorship-bias sensitivity analysis could not be performed. Despite these limitations, the database was harmonized and standardized, and analyses were performed on available cases with transparent reporting of the number of observations included in each comparison.

## 5. Conclusions

The analyzed cohort (n = 87) describes a clinical population with acromegaly characterized by a predominance of diagnosis in middle adulthood and a female predominance, in whom tumor features at diagnosis were frequently advanced, as reflected by the high prevalence of macroadenomas, extrasellar extension, and a notable proportion of giant adenomas. Management was multimodal in most cases, with surgery as the main intervention, frequently complemented by radiotherapy and maintenance medical therapy, in accordance with the tumor profile and the need for long-term disease control.

The therapeutic interventions applied resulted in a significant and robust longitudinal reduction in insulin-like growth factor 1, confirming the overall effectiveness of the treatment strategies used. However, sustained remission was documented in a relatively small proportion of patients, and a substantial number remained in the category of partial response, underscoring the chronic nature of the disease and the need for continuous treatment adjustment. The clinically relevant proportion of lack of response to first-generation somatostatin receptor ligands supports the importance of therapeutic escalation strategies and provides a clinical rationale for further exploration of the biological mechanisms underlying treatment response heterogeneity.

Overall, within the limitations of a single tertiary-center inpatient cohort, these findings support the view of acromegaly as a disease that is often recognized late in terms of tumor stage and requires multidisciplinary management and long-term monitoring, with integration of biochemical markers, clinical status, and imaging findings into therapeutic decision-making.

## Figures and Tables

**Table 1:** Medical treatment regimens at the last available evaluation.

Treatment Regimen	n	%
No medical treatment	42	48.3
Dopamine agonists	10	11.5
First-generation SRLs	18	20.7
Second-generation SRLs	3	3.4
GH receptor antagonist	1	1.1
Dopamine agonists + first-generation SRLs	9	10.3
Dopamine agonists + second-generation SRLs	1	1.1
First-generation SRLs + GH receptor antagonist	1	1.1
Dopamine agonists + second-generation SRLs + GH receptor antagonist	2	2.3
Total	87	100.0

Abbreviations: SRLs, somatostatin receptor ligands; GH, growth hormone.

**Table 2:** Hormonal profile and sex-based comparisons.

Variable	Comparison	Result	Statistical Test	*p*-Value
GH during OGTT	Women vs. men at diagnosis	Women: 16.12 ± 14.03 ng/mL; Men: 12.99 ± 10.98 ng/mL	t(50) = 0.79	0.434
GH during OGTT	Women vs. men at the first available evaluation	Women: 16.17 ± 13.95 ng/mL; Men: 11.86 ± 10.39 ng/mL	Welch t(46.15) = 1.33	0.189
IGF-1	Women vs. men at diagnosis	Women: 603.3 ± 249.8 ng/mL; Men: 734.4 ± 418.3 ng/mL	Welch t(25.23) = −1.30	0.205
IGF-1	Women vs. men at the first available evaluation	Women: 600.6 ± 254.1 ng/mL; Men: 774.3 ± 417.5 ng/mL	Welch t(27.95) = −1.81	0.081
IGF-1	First available evaluation vs. last evaluation	Lower at last evaluation in 68/73 patients; higher in 4/73; unchanged in 1/73; large effect size (r = 0.85)	Wilcoxon signed-rank test, Z = −7.25	<0.001

**Table 3:** Comorbidities and associated complications in the study cohort.

Comorbidity	n	%
Dyslipidemia	54	62.1
Nodular goiter	48	55.2
Arterial hypertension	47	54.0
Obesity	42	48.3
Ocular disorders	40	46.0
Gallbladder lithiasis	32	36.8
Other gastroenterological disorders	28	32.2
Gynecological disorders *	18	30.0
Musculoskeletal/orthopedic involvement	26	29.9
Benign prostatic adenoma/hyperplasia **	8	29.6
Diabetes mellitus	24	27.6
Hepatic steatosis	21	24.1
Renal cysts	19	21.8
Osteopenia	17	19.5
Sleep apnea syndrome	14	16.1
Prediabetes	14	16.1
Empty sella	13	14.9
Osteoporosis	13	14.9
Arrhythmias	12	13.8
Ischemic history	11	12.6
Anxiety–depressive syndrome	11	12.6
Autoimmune thyroiditis	11	12.6
Chronic infectious diseases	10	11.5
Primary hypothyroidism	8	9.2
Renal involvement	8	9.2
Hernias	8	9.2
Adrenal adenoma	7	8.0
Bradycardia	6	6.9
Neoplasms	6	6.9
Hepatic cysts	6	6.9
Dolichocolon	5	5.7
Disc herniation	5	5.7
Other intracranial abnormalities	5	5.7
Renal lithiasis	5	5.7
Severe aortic involvement	4	4.6
Colonic diverticulosis	4	4.6
Reactive hypoglycemia	3	3.4
Papillary thyroid carcinoma	3	3.4
Nasal septum deviation	3	3.4
Parathyroid disease	3	3.4
Colonic polyps	2	2.3
Pituitary apoplexy	2	2.3
Epilepsy	2	2.3
Intestinal obstruction	2	2.3

* Percentage calculated among women. ** Percentage calculated among men.

**Table 4:** Prevalence of pituitary insufficiencies at diagnosis, during follow-up, and at the last available evaluation.

Axis/Insufficiency Type	At Diagnosis, n (%)	During Follow-Up, n (%)	At the Last Available Evaluation, n (%)
Thyrotropic insufficiency	9 (10.3)	32 (36.8)	30 (34.5)
Gonadotropic insufficiency, complete	9 (10.3)	28 (32.2)	32 (36.8)
Gonadotropic insufficiency, partial	0 (0.0)	2 (2.3)	1 (1.1)
Corticotropic insufficiency, complete	3 (3.4)	16 (18.4)	15 (17.2)
Corticotropic insufficiency, partial	1 (1.1)	13 (14.9)	13 (14.9)
Somatotropic insufficiency	1 (1.1)	4 (4.6)	4 (4.6)
Diabetes insipidus	0 (0.0)	10 (11.5)	2 (2.3)

## Data Availability

The data presented in this study are available on reasonable request from the corresponding author. The data are not publicly available due to privacy and ethical restrictions.

## Abbreviations

The following abbreviations are used in this manuscript: GH, growth hormone; IGF-1, insulin-like growth factor 1; IMRT, intensity-modulated radiation therapy; LAS, Liege Acromegaly Survey; MRI, magnetic resonance imaging; OGTT, oral glucose tolerance test; SRLs, somatostatin receptor ligands; VMAT, volumetric modulated arc therapy.
